# H3ABioNet genomic medicine and microbiome data portals hackathon proceedings

**DOI:** 10.1093/database/baab016

**Published:** 2021-04-17

**Authors:** Faisal M Fadlelmola, Kais Ghedira, Yosr Hamdi, Mariem Hanachi, Fouzia Radouani, Imane Allali, Anmol Kiran, Lyndon Zass, Nihad Alsayed, Meriem Fassatoui, Chaimae Samtal, Samah Ahmed, Jorge Da Rocha, Souad Chaqsare, Reem M Sallam, Melek Chaouch, Mohammed A Farahat, Alfred Ssekagiri, Ziyaad Parker, Mai Adil, Michael Turkson, Aymen Benchaalia, Alia Benkahla, Sumir Panji, Samar Kassim, Oussema Souiai, Nicola Mulder

**Affiliations:** Centre for Bioinformatics and Systems Biology, Faculty of Science, University of Khartoum, Al-Gamaa Ave, Khartoum 11115, Sudan; Laboratory of Bioinformatics, Biomathematics and Biostatistics (BIMS), Institut Pasteur de Tunis (IPT), 13, Place Pasteur BP 74, Tunis 1002, Tunisia; Laboratory of Biomedical Genomics & Oncogenetics, Institut Pasteur de Tunis, Université Tunis El Manar, 13, Place Pasteur BP 74, Tunis 1002, Tunisia; Laboratory of Bioinformatics, Biomathematics and Biostatistics (BIMS), Institut Pasteur de Tunis (IPT), 13, Place Pasteur BP 74, Tunis 1002, Tunisia; Faculty of Science of Bizerte, University of Carthage, Zarzouna, Bizerte 7021, Tunisia; Research Department, Chlamydiae and Mycoplasmas Laboratory, Institut Pasteur du Maroc, 1, Place Louis Pasteur, Casablanca 20360, Morocco; Laboratory of Human Pathologies Biology, Department of Biology, Faculty of Sciences, Mohammed V University, 4 Ibn Battouta Avenue, Rabat, BP 1014 RP, Morocco; Genomic Center of Human Pathologies, Faculty of Medicine and Pharmacy, Mohammed V University, Rabat, Morocco; Malawi-Liverpool Wellcome Trust, Clinical Research Programme, PO Box 30096, Chichiri, Blantyre 3, Blantyre, Malawi; Computational Biology Division, N1.05 Werner Beit North, Department of Integrative Biomedical Sciences, Faculty of Health Sciences, Anzio Road, Observatory, Cape Town 7925, South Africa; Centre for Bioinformatics and Systems Biology, Faculty of Science, University of Khartoum, Al-Gamaa Ave, Khartoum 11115, Sudan; Laboratory of Biomedical Genomics & Oncogenetics, Institut Pasteur de Tunis, Université Tunis El Manar, 13, Place Pasteur BP 74, Tunis 1002, Tunisia; Faculty of Sciences Dhar El Mahraz, Department of Biology, Genetics Unit, Atlas-Fez 1796, Morocco; Centre for Bioinformatics and Systems Biology, Faculty of Science, University of Khartoum, Al-Gamaa Ave, Khartoum 11115, Sudan; Sydney Brenner Institute for Molecular Bioscience, University of the Witwatersrand, 9 Jubilee Road, Parktown, Johannesburg 2193, South Africa; National Institute of Health, Informatics Unit, 27 Ibn Batouta Avenue, Agdal, Rabat BP 769, Morocco; Medical Biochemistry & Molecular Biology, Faculty of Medicine, Ain Shams University, Abassia, Cairo 11381, Egypt; Department of Basic Medical Sciences, Faculty of Medicine, Galala University, Galala City, Suez 43511, Egypt; Laboratory of Bioinformatics, Biomathematics and Biostatistics (BIMS), Institut Pasteur de Tunis (IPT), 13, Place Pasteur BP 74, Tunis 1002, Tunisia; Information Systems Department, Faculty of Computers and Artificial Intelligence, Helwan University, Ain Helwan, PO Box 11795, Cairo, Egypt; Division of Entomology and core molecular Biology/Bioinformatics facility, Uganda Virus Research Institute, 51/59, Nakiwogo Road, Entebbe 31301, Uganda; Computational Biology Division, N1.05 Werner Beit North, Department of Integrative Biomedical Sciences, Faculty of Health Sciences, Anzio Road, Observatory, Cape Town 7925, South Africa; Centre for Bioinformatics and Systems Biology, Faculty of Science, University of Khartoum, Al-Gamaa Ave, Khartoum 11115, Sudan; National Institute for Mathematical Sciences, PMB Kwame Nkrumah University of Science and Technology (KNUST), Kumasi, Ghana; Laboratory of Bioinformatics, Biomathematics and Biostatistics (BIMS), Institut Pasteur de Tunis (IPT), 13, Place Pasteur BP 74, Tunis 1002, Tunisia; Laboratory of Bioinformatics, Biomathematics and Biostatistics (BIMS), Institut Pasteur de Tunis (IPT), 13, Place Pasteur BP 74, Tunis 1002, Tunisia; Computational Biology Division, N1.05 Werner Beit North, Department of Integrative Biomedical Sciences, Faculty of Health Sciences, Anzio Road, Observatory, Cape Town 7925, South Africa; Medical Biochemistry & Molecular Biology, Faculty of Medicine, Ain Shams University, Abassia, Cairo 11381, Egypt; Laboratory of Bioinformatics, Biomathematics and Biostatistics (BIMS), Institut Pasteur de Tunis (IPT), 13, Place Pasteur BP 74, Tunis 1002, Tunisia; Computational Biology Division, N1.05 Werner Beit North, Department of Integrative Biomedical Sciences, Faculty of Health Sciences, Anzio Road, Observatory, Cape Town 7925, South Africa

## Abstract

African genomic medicine and microbiome datasets are usually not well characterized in terms of their origin, making it difficult to find and extract data for specific African ethnic groups or even countries. The Pan-African H3Africa Bioinformatics Network (H3ABioNet) recognized the need for developing data portals for African genomic medicine and African microbiomes to address this and ran a hackathon to initiate their development. The two portals were designed and significant progress was made in their development during the hackathon. All the participants worked in a very synergistic and collaborative atmosphere in order to achieve the hackathon's goals. The participants were divided into content and technical teams and worked over a period of 6 days. In response to one of the survey questions of what the participants liked the most during the hackathon, 55% of the hackathon participants highlighted the familial and friendly atmosphere, the team work and the diversity of team members and their expertise. This paper describes the preparations for the portals hackathon and the interaction between the participants and reflects upon the lessons learned about its impact on successfully developing the two data portals as well as building scientific expertise of younger African researchers.

**Database URL**: The code for developing the two portals was made publicly available in GitHub repositories: [https://github.com/codemeleon/Database; https://github.com/codemeleon/AfricanMicrobiomePortal].

## Introduction

There is a bias in public genomic databases toward data from European and North American populations, and most of the public genomic databases have just a few datasets from the African continent (1). African genomic medicine and microbiome datasets are usually not well characterized in terms of their origin, making it difficult to find and extract data for specific African ethnic groups or even countries. The Pan-African H3Africa Bioinformatics Network (H3ABioNet) recognized the need to address this by developing two online web portals: (i) African Genomic Medicine Portal (AGMP) and (ii) African Microbiome Portal (AMP) to provide links to curated African data in public databases. In order to progress the design and development of these portals, H3ABioNet agreed to support the recruitment of participants across its nodes in several African countries to participate in a portal development hackathon. H3ABioNet has previously organized two hackathons, one on the Malaria Drugs DREAM challenge (2) and another aimed at developing bioinformatics workflows (3, 4).

The main objectives of this third wave of H3ABioNet hackathons were to design and develop two portals, for African Genomic Medicine and African Microbiome studies, and to curate and harmonize publicly available data for these databases.

Hackathons are intense, short, collaborative events in which participants with expertise in the domains of software engineering and biomedical research come together to work intensely over 3–6 days focused on creating innovative solutions for pressing problems (5). Recently, hackathons have gained popularity in the bioinformatics community, offering considerable potential for innovation in global health based on local needs and resources as well as addressing feasibility and cultural contextualization (6). Bioinformatics hackathons more closely resemble a scientific discussion and provide an opportunity to learn and plunge into explicit and specific goals. A successful hackathon requires participants with both programming skills and domain-specific knowledge (e.g. genomic medicine and microbiome in our case). While these events have shown some success in the development of prototypes and healthcare technology solutions, one of their underappreciated successes has been as educational and training tools (7, 8).

H3ABioNet has a strong capacity development remit, includes members with a broad range of skills and expertise, and identifies a need to collaboratively develop two data portals for genomic medicine and microbiome research in Africa. A web portal represents a web site that provides a single point of access to applications and data to support effective information search and analysis as well as to enhance communication and collaboration among researchers in various scientific fields (9, 10).

This paper discusses the H3ABioNet efforts to bring together African scientists to design and develop the H3ABioNet African Microbiome Portal and the AGMP, specifically to accelerate data content curation, harmonization and development of these two portals to produce valuable resources that will be useful in the African context. We report on our experience running hackathons and explore the various issues of relevance to developing these two web-based portals.

## Methods

### Pre-hackathons preparation

H3ABioNet agreed to support the recruitment of participants across its nodes in several African countries to participate in this hackathon. Monthly meetings held by conference call for organizing the hackathon started in December 2018 continued until February 2019. Thereafter, two meetings were held per month until the date of the first hackathon.

A team of H3ABioNet leaders and junior academics that were active members in both projects and demonstrated their interest and commitment to the projects were selected. An official call for applications for hackathon participation was placed online in January 2019.

**Figure 1.**
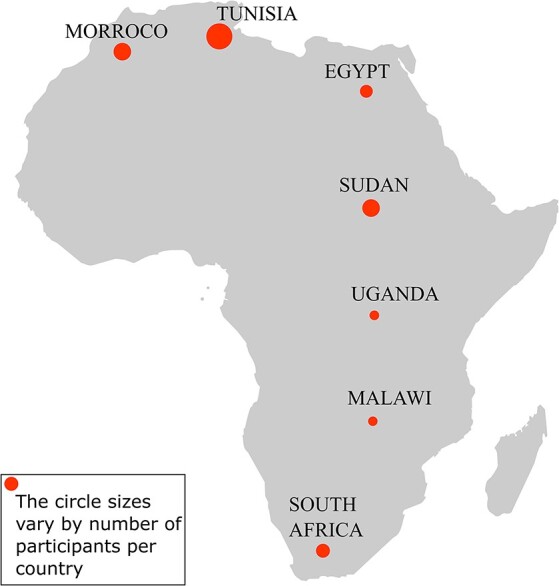
Participants’ representation by country in the hackathon. The hackathon involved 24 participants from seven African countries: Tunisia (8), Morocco (4), Sudan (4), South Africa (3), Egypt (3), Uganda (1) and Malawi (1).

A committee from H3ABioNet reviewed 31 applications including 12 members involved in the genomic medicine portal project, 9 members involved in the microbial portal project and 10 members involved in both projects. The selection was mainly based on the levels of their involvement in both projects and/or their skills in web development that were assessed by an online questionnaire. The participant skills include technical skills in Python; Django; CSS and web programming; database development, deployment and maintenance; java; C++; MySQL; data/text mining; and data curation. The selected members originated from seven distinct African countries (Figure 1).

**Figure 2.**
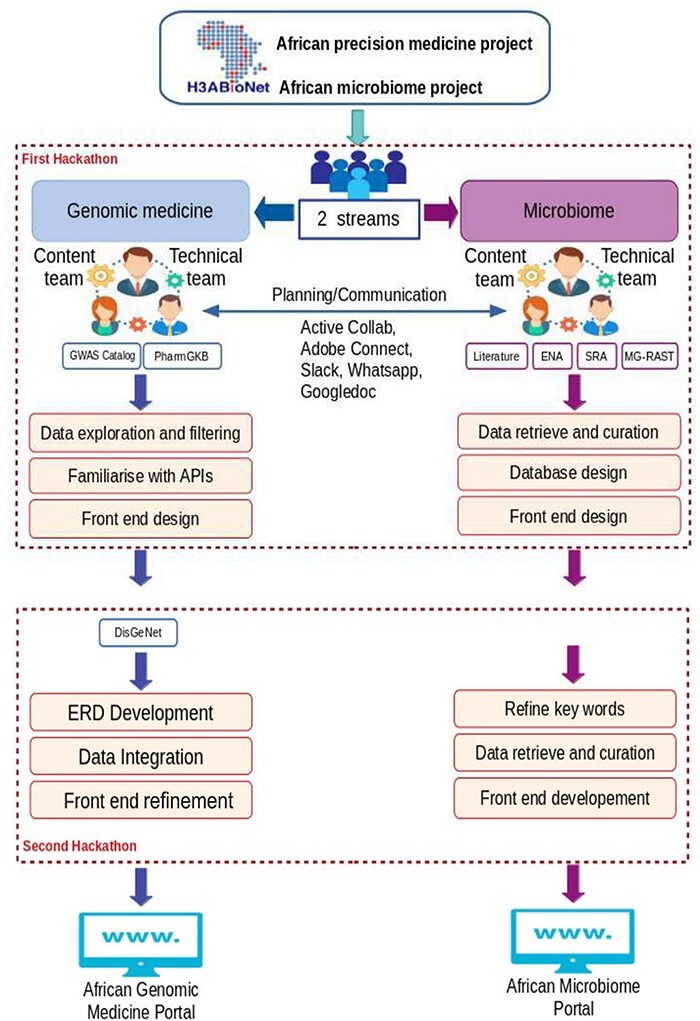
Main hackathons’ goals and components, including communication platforms.

Once the selection was made, online fortnightly meetings took place before the hackathon to initiate the discussion between all participants and to develop use-case scenarios for both portals. The main advantage of this pre-hackathon phase was that the team members started to collaborate with each other and identify data sources and types of data to be collected as well as the programming languages that would be used for portal interface development. Several communication and collaboration tools were used (including Adobe Connect for video conferencing and ActiveCollab) for exchanging ideas and sharing minutes of meetings and documents. A summary of the main hackathons’ goals and components, including communication platforms, is shown in Figure 2.

**Figure 3.**
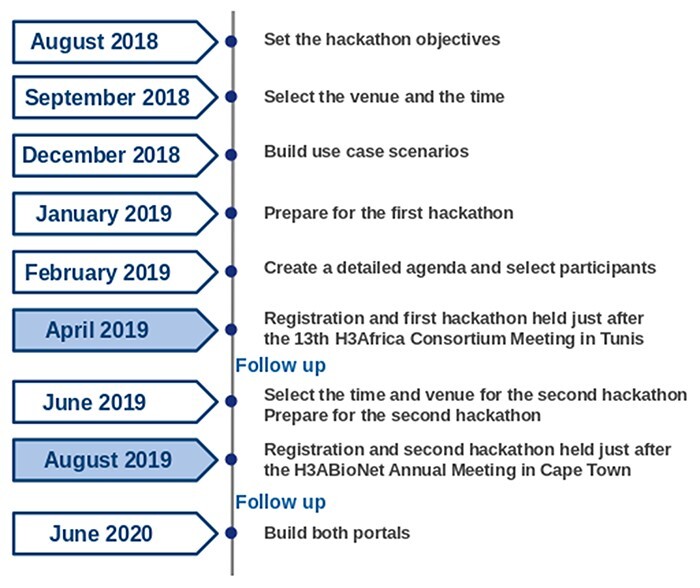
A timeline of the hackathons’ planning activities.

A summary of the two hackathons’ timeline planning is provided in Figure 3.

All the hackathon participants were H3ABioNet and H3Africa consortium members, who have an element of genomic medicine and/or microbiome research in their project’s objectives and were able to contribute to the outcomes of the hackathons. They were selected based on their diverse scientific backgrounds, including computer science, bioinformatics and biology. Among the participants, some displayed strong skills in database and web application, portal development, and computer science, while others had knowledge of metadata curation with no or minimal experience in web development or computing. The participants were also at various stages of their careers, including MSc and PhD students, postdoctoral researchers, research associates and university faculty. Based on their background and research areas, the hackathon’s participants were grouped into two topic streams: Stream A for the African Genomic Medicine web portal and Stream B for the African Microbiome web portal. Both streams contained members with diverse and complementary areas of expertise, which created an ideal work environment to achieve the goals by combining their experiences. These could not have been achieved by individual members. An additional team in charge of writing and drafting a manuscript about the hackathon proceeding was also formed. Each stream consisted of technical members with programming skills as well as portal content experts, creating multidisciplinary teams to work in parallel on the different aspects of portal development. All the participants agreed to have a three-level testing approach for both portals. At the first level, the project members would switch the portals between them to be tested by members of the other project. Portals will then be shared with all of the H3ABioNet members for the second-level testing and feedback will be captured. For Level 3, portals will be shared with H3Africa Principal Investigators (PIs) for final testing and feedback.

### Hackathon proceeding and activities

The first hackathon was held in April 2019 at Institut Pasteur de Tunis, Tunisia, following the 13th Meeting of the H3Africa Consortium organized in Tunis. The hackathon program was based on a series of presentations and talks, highlighting the objectives of both portals during the first day and portal design and development breakout group sessions from the second to sixth day. In addition, the first day of the hackathon was also dedicated to a detailed presentation of the use-case scenarios for both portals, the different aspects of data that need to be integrated in both portals and how the output should be displayed to users. This was followed by a discussion in each stream about the planning of the week. The hackathon was an occasion to bring together H3ABioNet project members involved in the design, conception and development of the two portals.

Teams then split into their individual collaboration areas at Institut Pasteur de Tunis, to plan their data collection, curation, harmonization and integration strategies. At the end of each day, a report-back session was scheduled, during which each of the teams presented their progress and challenges faced by participants. This generated interesting open group discussions that provided the different teams with constructive feedback from all participants. This helped teams to crystallize ideas, merge efforts and refine strategies. A daily working plan was prepared the day before, and each day’s progress was presented and discussed with the H3ABioNet central node represented by the consortium PI and the network project manager.

Although the two different streams achieved significant progress toward data curation, cleaning, harmonizing, web page design, portal tutorial development and database development during the hackathon, further work was required for both the portals. The team members committed to contributing and investing time to finalize the development after the hackathon. On the last day of the first hackathon, each team presented a final project plan along with a timeline.

A second hackathon took place after the H3ABioNet annual general meeting in August 2019 in Cape Town, South Africa, where a few members of the two projects met for one week to refine both portals in terms of the contents and to add and refine portal features and functionalities.

#### African Genomic Medicine Portal (AGMP)

During the first hackathon, the genomic medicine content team explored various databases from which data could be retrieved using Application Program Interface (API). These included PharmGKB, ClinVar, GWAS Catalog, dbSNP, BioMart, OMIM, ClinGen, VIP, Monarch Initiative, MyVariant, PharmVar and others. They followed a protocol to evaluate the most informative databases to be used to extract African data and identify metadata to be included. There was also a focus on the design of these databases to determine the processes needed to retrieve and curate consistent data that will be incorporated into the AGMP. The team also aimed to identify databases, which already included ethnicity or geographical region-related data that could be easily maintained.

The content team was not experienced in API use, therefore they worked continually with the technical team to progress and address their needs. The steps they followed were as follows:

- Familiarize themselves with APIs;
- Identify databases from which information could be retrieved using APIs;
- Identify the information in databases and what information they need;
- Design search outputs;
- Develop and refine data filters;
- Set the needs from the technical team (portal interface design, data retrieval, data output, etc.).

Ultimately, two databases were selected for initial information incorporation into the portal, namely PharmGKB and the GWAS Catalog. These databases were selected because of the classification of data records already incorporated within them, enabling African-related records to be easily retrieved.

Contextual filters were then set to retrieve African data from the two databases; however, it was later noticed that a lot of African data were annotated as larger or unclassified regions and ethnic groups. Therefore, the content team opted instead to employ a manual mining approach to extract African-specific data. Another proposed filtering approach was through using PubMed Identifiers.

**Figure 4.**
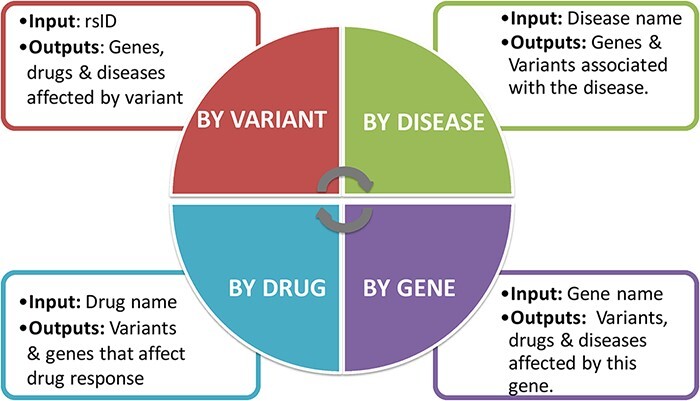
Four different search options have been added to the interface: searching by variant, gene, disease and drug.

The portal was designed to have a multi-search functionality, with data related to genes, variants, diseases and drugs. The proposed search functionality is illustrated in Figure 4.

##### PharmGKB.

PharmGKB is a publicly available database that contains information regarding the impact of human genetic variation on drug response. It was, therefore, selected to provide data on drug processing associated with genetic variation in African populations through the portal. It inherently classifies relevant data as Sub-Saharan African, African-American and Near Eastern (11).

##### GWAS Catalog.

GWAS Catalog is a publicly available database that contains information regarding published genome-wide association studies. This database classifies relevant data as Sub-Saharan African, African-American and Greater Middle Eastern (12).

During the second hackathon, the AGMP team switched the design to a static relational database design because of significant challenges that were faced in the automated retrieval of data through APIs. It was evident that some data curation was required. Thus, an Entity Relationship Diagram (ERD) was designed and implemented in the portal backend. During this second hackathon, the portal interface was further designed and refined. In addition, the content team decided to switch from GWAS Catalog to DisGeNET as the latter includes GWAS Catalog data in addition to many other data sources.

##### DisGeNET.

DisGeNET is a discovery platform that contains a comprehensive catalog of genes and variants associated with human diseases. Variant-disease information available in DisGeNET originates from ClinVar, the GWAS Catalog, UniProt, GAD and BeFree data (13).

#### African Microbiome Portal (AMP)

The African microbiome data collection included human metadata that were retrieved from public repositories, namely, the metagenomics RAST server, European Bioinformatics Institute Metagenomics platform and the Sequence Read Archive (14, 15, 16) as well as from a microbiome literature repertoire retrieved from PubMed by the microbiome content team prior to the hackathon. Both content and technical team members engaged in intense collaboration during the hackathon through a series of brainstorming and discussion sessions, and as a result, various issues were addressed, clearly defined and efficiently tackled in a short period of time.

During the first hackathon, most of the content work was devoted to harmonizing and refining of data with the metadata scope being extended to include entries that are entirely relevant to the samples and technical details from each project-related publication. In addition, portal graphical web interface design and the main and advanced query search options, along with the conceptual model of relational database schema development, were discussed and achieved by both the technical and content teams. The team has stressed that the hackathon outcomes provided an excellent starting point to drive the database implementation post hackathon. A timeline was set to keep track of the team activities to achieve the project goals. In addition, subtasks were assigned to different members, aiming at better coordination.

## Results

The hackathons provided an opportunity for the participants to meet face to face and discuss some H3ABioNet tasks and activities they were involved in. The following subsections describe the tasks and activities the hackathon participants managed to complete during and after the hackathons.

**Figure 5.**
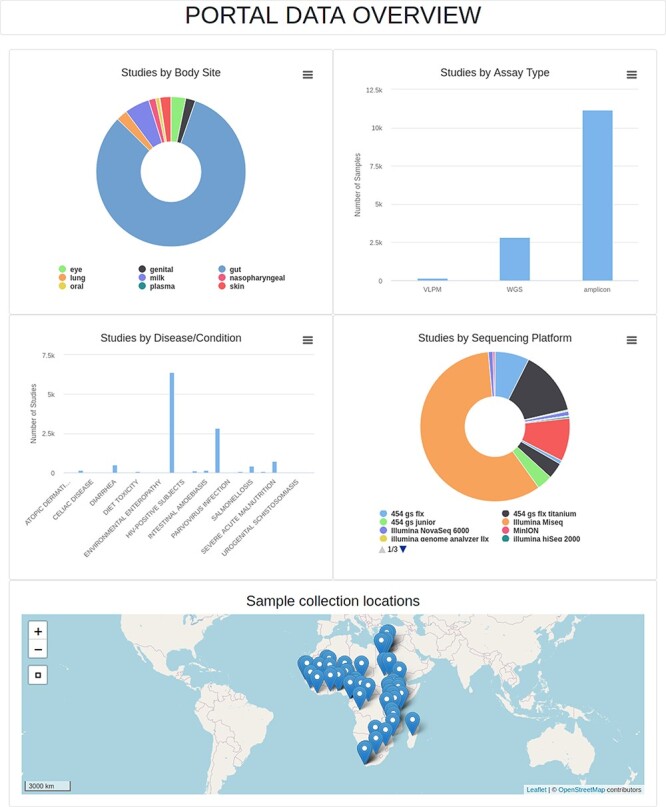
Data overview page of the AMP. VLPM: Virus-like particle metagenomics; WGS: Whole-genome sequencing.

**Figure 6.**
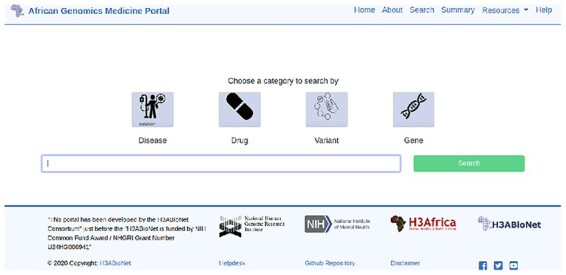
Search page of the AGMP.

Members of each project drafted a use-case scenario for their portal, specifications for data formats and a representative interface design before the hackathon. This information guided the technical team through their progressive portal development and updates. The continued dialog between the technical and content teams (during meetings and other online platforms) has enabled the refinement of the database schemas, models and the interface. The portals are both built on the Django framework with MySQL/SQLite database backend. The interface has been developed using HTML, CSS and JavaScript library Bootstrap4. Briefly, the portals have search functionality that retrieves results with hyperlinked details arranged in collapsible tabular formats, a summary page with interactive hyperlinked visual summaries of the data and a map showing the distribution of these data on the African continent with pop-ups containing fundamental details for each mapped record. Figure 5 shows a snapshot of the AMP data overview. AMP enables data upload via a user interface, however this will be only accessible by portal administrators. Similarly, Figure 6 shows a snapshot of the AGMP search page.

Data curated by the content team are shared with the technical team in spreadsheets. These sheets are then ingested into the respective databases. Other metadata are added to the curated data in order to relate records in a proper relational manner.

Preliminary testing of an early version of the system by members of the respective work groups uncovered issues in both curated data and the application. Issue boards were set up for collecting such feedback, while improvements and fixes were made in an iterative manner.

### Genomic medicine content team outcomes

In addition to extracting content, the genomic medicine working group members had the opportunity to discuss other tasks they were developing, such as the progress on a pharmacogenomics review paper they were drafting (1). They established the paper design and settled on the types of data to be included. Furthermore, they launched the Omics review manuscript, which was an outcome of the discussion during the data extraction and the gaps encountered during the first hackathon.

At the end of the hackathon, the members finished by agreeing on the portal’s interface forms and the information and data to include. Members also agreed on how to approach the filtering of data and data extraction and developed a plan to monitor the implementation of the portal. The genomic medicine content team also set different tasks [portal tutorial, automated metadata filtering process, finalization of the review paper (1) and starting the Omics review manuscript] and assigned members to coordinate these tasks to ensure progress after the implementation, as well as setting a working plan with the technical team to follow the finalization of the portal implementation.

### African microbiome content team outcomes

The content team felt that the hackathon provided an excellent starting point to drive the database implementation post hackathon. The metadata were refined and allowed a better sensitivity regarding the portal scope. Indeed, the undertaken manual data curation and harmonization process provided an initial dataset to set up a preliminary version of the portal. In addition, the web portal template gave a first overview of the different functionalities of the portal and guided the technical team on how to design the portal’s web interface.

### Data portal design and development

Selection of a broad community-supported web framework is essential for a service’s sustainability. It is also important to identify the language preference of programmers in the organization. At the first hackathon in Tunis, Java–Spring Boot, Python–Django and PHP–Drupal web frameworks were recommended by technical team members as their choices. Java–Spring Boot was selected based on its initial recommendations, as a website template was provided by H3ABioNet. However, due to inconsistencies in Spring Boot versions and the absence of Java or Spring Boot experts in the team, after consultation with the members, the Python–Django option was adopted, as most of the members were already using Python for their bioinformatics tasks and it is widely adopted in H3ABioNet. The graph database management system Neo4j was initially proposed as a data storing system for the AGMP, given the complex relationships that existed among different components of the database, while a relational database was used for the AMP, populated with dummy data (as supplied by the content team). For the web interface with basic search functionality, Bootstrap 4 was used. Portals with basic functionalities were demonstrated at the end of the hackathon. Moving forward, the team explored features such as auto completion, advanced search, data visualization and Google alerts for data curation, as well as documentation of the design and how to use the portals.

### Second data portal hackathon

Between the first and second hackathons, the AGMP team faced significant challenges regarding the automated retrieval of data using APIs as previously outlined. This included missing information and technical challenges. Therefore, it was decided to switch to a static database design. With this approach, data would be retrieved from the selected databases and manually curated by a content curation team, which involved a process of considerable text mining of the respective websites and online databases. The content team also decided to switch from the GWAS Catalog to DisGeNET, because DisGeNET contained information from a wider variety of sources, including the GWAS Catalog. During the second hackathon, an ERD was designed and implemented in the portal backend. In addition, the portal interface was further designed and refined.

The data portal resources are not yet online; they are in the testing phase and will be publicly available soon. Therefore, no stable links to the portals are available yet; however, links to the GitHub repositories and code used for both portals are available.

### Challenges of the hackathon

The content and technical teams of both portals had to face more or less the same challenges during the hackathon. The following are the common challenges faced:

1. Content team members faced some challenges when filtering African data. For example, sometimes the retrieved information was not cited as specific to African populations (e.g. African-American, Mixed population, Near East and Greater Middle East). To remedy these issues, the content team members opted for manual data curation after the automatic process of filtration—this allowed the team to confirm and curate the retrieved information before inclusion in both portals.
2. The content team members had to exclude some databases even though they have valuable information because they did not have APIs.
3. Some databases (e.g., OMIM) needed permission to access their APIs.
4. Some Internet connection issues occurred during the hackathon, which slowed down progress, even though the work progressed and goals were reached.
5. There was an absence of Java or Spring Boot experts within the technical team.

The lack of African metagenomic metadata in public repositories has pushed the microbiome content team to extend the search to PubMed. At the time of the hackathon, it was decided to include additional information in order to give a more complete description of the samples. However, matching the project to its corresponding publications was also difficult. In addition, manual curation of the metadata in an Excel file with thousands of lines was a laborious and time-consuming process.

### Hackathon feedback

**Figure 7.**
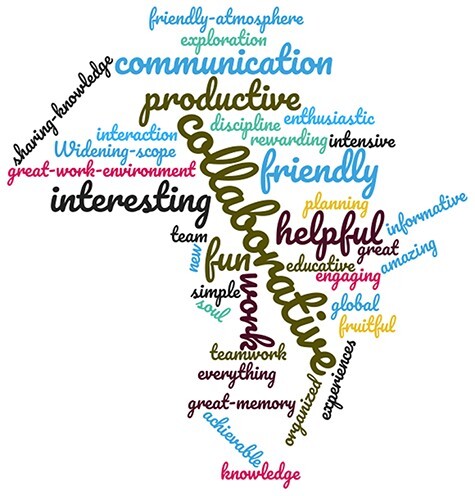
The hackathon as seen by the participants. The figure was generated using https://www.wordclouds.com.

After the hackathon, the organizing committee sought feedback from the participants on what they thought of the event, how they found the atmosphere and the process, and what they learned during the hackathon, as well as what they learned within a year following the hackathon. The organizing committee developed a survey consisting of a few simple questions; here, we summarize the participants’ feedback. Figure 7 highlights the words used by the participants to describe the event. When asked what they learned during the 1-week event, participants’ answers differed based on their background and whether they were familiar with computer science or not. For the content team members, participants gained new knowledge on how to design and implement databases, how to explore existing databases and how to set up filters to retrieve consistent information. The technical team members were introduced to Django as a high-level Python Web framework, Java, NoSQL and Neo4j as a robust graph database platform in addition to a few aspects of Spring Boot as a web development platform. They mainly learned how to interact with database APIs to fetch and retrieve information and how to work collaboratively as a team for the design and implementation of both portals.

In response to a question on what participants liked the most during the hackathon, 55% highlighted the familial and friendly atmosphere, the teamwork, and the diversity of team members and their expertise. Participants also encountered some challenges related to Internet connection issues that did not affect the progress of the work and achieving the goals. Twenty-five percentage of participants preferred that the hackathon would be longer than one week. Technical team members highlighted some problems regarding the delay in choosing the programming languages that will be used for portal design and implementation. In order to assess knowledge transfer after the hackathon, all participants were asked to provide details about what they learned one year after the hackathon. Thirty percentage of the answers corresponded to the acquisition of knowledge regarding technical aspects and skills on portal development and design, supporting the idea of knowledge transfer between participants. The other answers were diverse including learning how to harmonize metadata, teamwork and work planning (See Supplementary file S1). Since the hackathon, participants continued to work as a team and collaborate to finalize both portals. Participants continued to learn and consolidate the knowledge and skills acquired during the hackathon. Since the hackathon, some participants were able to participate in other hackathons. Finally, participants were asked to list what kind of events the hackathon helped them to organize one year later. For nearly 30% of the participants, the hackathon helped them to run training courses, workshops, webinars, other hackathons on social engagement and social behavior, as well as a virtual hackathon on OMICs data analysis. Gathering this feedback is very useful for hackathon organizers to understand how the event was perceived by the participants and to better plan for future similar events.

### Lessons learned

Advantages of the hackathon and lessons learned include attention to subjects that are pertinent to the participants, an opportunity for cooperative advancement, adaptability of timetable and commitments from each member. The connections developed during a hackathon frequently continue well past the event. The communications can promote profitable coordinated efforts and are efficient at building a network between members. Group work and interactivity were the basis of these hackathons.

## Discussion

In recent years, there has been a growing movement to use hackathons to bring multidisciplinary teams together to generate excitement and momentum around collaborative projects and demonstrate what can be accomplished when the right partners are at the table (6).

Hackathons are valuable in bringing domain specialists and specialized computer scientists with different degrees of involvement and aptitudes to ‘hack’ solutions to scientific questions. Whereas conventional conferences focus on exchanging information, hackathons are more collaboratively producing and generating solutions (17). The interactions often lead to professional development opportunities, a network of resources and profitable collaborations. Actually, we found that the H3ABioNet portal hackathon participants were more interested in working in collaborative projects after the hackathon than before.

The H3ABioNet data portal hackathon was aimed at producing an AGMP and an AMP to fill the gap in these two fields with regard to African datasets.

One of the factors that contributed to the success of the hackathon is the respectful environment and friendly atmosphere where it was held (17). Other key factors contributing to the success of the hackathon include planning strategy and meetings before the hackathon week proved to be the backbone for a successful hackathon, productive results and fulfilling the objectives of the hackathon (17). In addition, regular fortnightly meetings after the hackathons helped to further refine the approach, add more metadata and refine the design of the interface of the portals. Moreover, adopting a well-defined communication approach made interactions among the participants easier in terms of sharing documents and ideas in real time (17).

Feedback from hackathon participants was for the most part positive, and the eagerness that participants felt was apparent during the hackathon. After the hackathon, the participants continued communicating around the portal work as well as contributing toward addressing African dataset gaps in the public databases. In addition, when responding to what they learned during the 1-week hackathon, participants’ feedback differed based on their backgrounds. The content team participants gained new basic knowledge on how to design and implement web-based portals and databases, whereas the technical team learned how to interact with database APIs to retrieve information and most importantly how to collaborate as a team for the design and implementation of the two portals.

We trust that the outputs of this hackathon assist the African biomedical research community, as they fill the gap through making the African datasets in genomic medicine and microbiome studies more easily findable. We encourage African scientists working in these two fields to deposit and submit their research findings into the developed portals and to assist with further evaluation of these two portals. Further research is needed to determine what the impact of the two portals will be within the wider biomedical research community. Further information with regard to the indicators we will use for the evaluation of these portals can be found in the ELIXIR Core Data Resources (18).

## Conclusions

Hackathons have been demonstrated to provide an excellent opportunity for scientists from different backgrounds (bioinformatics, computer science, genomic sciences, etc.), to work together over 6 days to thoroughly understand a specific question/problem and try their best to solve it utilizing a multidisciplinary and collaborative approach. The data portal hackathon gave members a new perspective on cooperation, which can change how they approach their everyday work afterward.

The AMP was developed to establish a centralized repository for microbiome metadata associated with African populations, while the AGMP was developed to provide a resource that collates African genomics data to facilitate the browsing of knowledge on the genetic underpinnings associated with disease and drug response in African populations. Both AGMP and AMP provide researchers a platform from which they can retrieve existing information and resources on their respective topics.

Preliminary testing of an early version of the two portals by members of the respective working groups uncovered issues in both curated data and the applications. Issue boards were set up for collecting the feedback, while improvements and fixes were made in an iterative manner. From our preliminary testing and evaluation, we determined that the hackathon was successful at fulfilling its goals of developing two data portals. Evaluation of the two portals is an ongoing process, which will continue even after release to the wider biomedical research community. Once version one of the portal will be released, we will first work on adding some additional features to the portal, such as informative graphs based on the data contained in the portals, exploring different methods of expanding the information integrated in the portals, investigating both a community-driven input method, and adding data from more online resources. Finally, in terms of sustainability, we are also exploring how we can collaborate with these existing resources to facilitate data curation and ingestion.

## Supplementary Material

baab016_Supp
