# Correction to: H3ABioNet genomic medicine and microbiome data portals hackathon proceedings

**DOI:** 10.1093/database/baad068

**Published:** 2023-10-13

This is a correction to: Faisal M Fadlelmola, Kais Ghedira, Yosr Hamdi, Mariem Hanachi, Fouzia Radouani, Imane Allali, Anmol Kiran, Lyndon Zass, Nihad Alsayed, Meriem Fassatoui, Chaimae Samtal, Samah Ahmed, Jorge Da Rocha, Souad Chaqsare, Reem M Sallam, Melek Chaouch, Mohammed A. Farahat, Alfred Ssekagiri, Ziyaad Parker, Mai Adil, Michael Turkson, Aymen Benchaalia, Alia Benkahla, Sumir Panji, Samar Kassim, Oussema Souiai, Nicola Mulder, H3ABioNet genomic medicine and microbiome data portals hackathon proceedings, Database, Volume 2021, 2021, baab016, https://doi.org/10.1093/database/baab016 In this manuscript, a correction has been made to author Mohammed A. Farahat’s name.

