# Supplementary material for: H3ABioNet genomic medicine and microbiome data portals hackathon proceedings
Source: Database (Oxford). 2021 Apr 17;2021:baab016. doi: 10.1093/database/baab016 (PMC8052916; doi:10.1093/database/baab016)
Supplement: baab016_Supp [file baab016_supp.zip › Supplementary_table_S1.pdf]

1. 1 What did you learned during the hackathon held on April 2019 in Tunis?	1.2 Describe the hackathon in few words (3 max)	1.3 Which team you belonged to?	1.4 In which portal development have you been involved?	1.5 What did you like the MOST about this hackathon?	1.6 What did you like the LEAST about this hackathon?	1.7 Any other comments/suggestions regarding the hackathon?	2.1 What did you learned from the hackathon one year after?	2.2 What has been the outcome of the hackathon on your work during this last year?	2.3 Did the hackathon helped you to organize similar events in your institution?	2.4 What kind of events did the hackathon helped you to organize during this last year?
During the hackathon, I learned lot: How design and implement data bases, How to explore existant database, how to extract existant information. we also build an area of complementarity between technical team (developers) and content team (biologist, physician...).	The hackathon was an opportunity for the working group members to meet face to face. Mainly to launch the why will implement the portal, and discuss it design and it's content. it was an opportunity for content team and technical to agree about their needs to make the goal ongoing. It was also an opportunity to make the work the start work processing and make set a plan for the upcoming activities	Both teams	African precision medicine portal	The working atmosphere that generated good productivity	Everything was good. However, I will mention the challenges in connection sometimes. This can happen in all circonstances, it was beyond the local organizers. Despite this, the work progressed and goals were reached.	I suggest to organize hackathons frequently this can make the work ongoing.<br>Thanks to the organizers and all participants	The progress of the work after the hackathon was consistant	The PM review paper was published, the white paper submitted, the Genomic Medicine portal is set, new database was used (DigeNet), lot of data extracted, curated and included to the portal, the finalisation of the portal is in progress. New plan to sustain the working group activities was set: Hackathon paper, Portal paper, precision medicine ebook, proposing new proposals, new members continually asking to join the PM working group, many other ideas are generated by the working group members, interaction and collaboration in a good atmosphere, ...	Yes	Courses, workshops, meeting to launch new activities
I learned about many of the technical considerations that need to be taken into account when developing a database. We explored different way of incorporating info into the database e.g. static vs API-based. We also explored several existing databases with genetic information, some of which I was not previously familiar with	collaborative, engaging, fun	Content team	African precision medicine portal	Getting the hands-on opportunity to work on the development of the portal with the members I typically only communicate with in online meetings. The hackathon provided the team an opportunity to work together and build team spirit, that pushed us forward in our work for the months following after the hackathon as well.	Too short	No	I have a lot more knowledge regarding the technical aspects of developing a database. I now work closely with the technical team in order to address issues that arise during development	The hackathon has driven the project members to work hard to complete a version 1 of the portal, the hackathon has built team spirit, the hackathon has inspired and allowed us to complete 2 manuscripts (one of which has been published).	No	None
Fine science, team work	Friends who work professionally	Content team	African precision medicine portal	This friendly atmosphere with high expertise guidance	Nothing	Thanks	Still friendly professional team work	Nearly finished portal	Yes	Training course
Fine science, team work	Friends who work professionally	Content team	African precision medicine portal	This friendly atmosphere with high expertise guidance	Nothing	Thanks	Still friendly professional team work	Nearly finished portal	Yes	Training course
I learnt a lot namely how to design a portal graphical interface and how to build it, How to develop the filters to extract the needed information in the existing explored databases, and how to extract API information.	team work soul	Content team	African precision medicine portal	the familial atmosphere and the team work soul and perseverance that every participant and organizer shows and that what was behind the success of the hackathon in my opinion	None	I guess if we had 2 more days we could have achieved more goals	I have now a good knowledge and skills on portals development and design, and I'm so grateful for that :)	it help me to know how to identify and extract the necessary metadata from the different existing databases and how to explore correctly these databases	No	None
Fine science, team work	Friends who work professionally	Content team	African precision medicine portal	This friendly atmosphere with high expertise guidance	Nothing	Thanks	Still friendly professional team work	Nearly finished portal	Yes	Training course
Portal development	intersting, friendly, fruitful	Content team	African precision medicine portal	I learned a lot from this Hackathon	It could have been better if it had lasted longer (very busy schedule)	Thanks to all participants and organizers	Portal development, data mining and portal interface design	It was the first time that I participate in a Hackathon, and since, I participated in two other Hackathons and I learned a lot on portal developments on data mining and portal interface design	Yes	a Hackathon on social engagement and social behaviour and a virtual Hackathon on OMICs data analysis in the framework of two Horizon 2020 projects .
- Learnt about Neo4j; it turns out to be very robust for databases with objects that have complex (and/or much more) relationships amongst them. It would rather be difficult to represent such data using relational database schemas.<br>- Learnt a few aspects of Spring boot as a web development platform. It was my first time trying it out. Even though, the technical team did not get on with it following a switch to Django, I still appreciated the need of good documentation of tools to ensure continuity of such projects.<br>-Lastly, I learnt a bit about usage of APIs to fetch data from publicly available databases, this was a trial of using APIs to fetch data from pharmaGkb. Though this approach was not taken on given some of its downsides, it was a great opportunity to experiment with it at the hackathon.	Interesting, Educative, Simple	Technical team	Both portals	- The team work amongst the participants in the different streams<br>- The flexibility of the core team to understand the concerns of the technical team. Specifically allowing the switch from spring boot to Django framework.<br>- The logistics in terms of accommodation and the conducive working environment.	Time spent on trouble shooting spring boot, it was a good initiative though did not turn	One comment about the tools that are developed, as developers, we need to ensure proper documentation of all the details to enable continuity in the future, it will save lots of time and resources.	Creating interactive graphical summaries for dynamic websites; these include maps, bar charts, pie charts among others.	Contribution to; implementation of the micro-biome portal dashboard, implementation of Django models and populating them with PharmaGKB and GWAS catalogue data for the African genomic medicine portal.	No	In my case, I have not prepared any events but we have an going project for which we are benefiting from the portal development side of the hackathon. In this project, we are analyzing metagenomics data hunting for viral pathogens, after the analysis, we shall create a portal to ease access and search of results in relation to sample data such as sampling location.
initial steps of relational database conception	collaborative, rewarding and productivity	Content team	African microbiome portal	mutual exchange of competences and the cooperation between the different members	Lack of activity with the different members after workdays	reduce the number of work hours and rather lengthen the duration of the hackathon	different technical aspects of the portal implementation	it was a real boost and allowed me to better approach my task within this project.	No	So far, I haven't been asked to organize any events.
I learned how to design a protocol to implement a portal	the Hackathon help us to make progress in the database conception and implementation, to share our knowledge with the group	Content team	African microbiome portal	Working in group	i would appreciate if the technical team share with us with details the approach used to implement the portal (sql, django, leaflet...) It was not the case	I suggest that the technical team communicate with details the methodology used to implement the portal	I learned how to harmonize metadata according to the recommendation of the technical team, to make progress on portal design and implementation	implementation of the portal, publications, valorization of this experience to implement databases	Yes	workshop

1. 1 What did you learned during the hackathon held on April 2019 in Tunis?	1.2 Describe the hackathon in few words (3 max)	1.3 Which team you belonged to?	1.4 In which portal development have you been involved?	1.5 What did you like the MOST about this hackathon?	1.6 What did you like the LEAST about this hackathon?	1.7 Any other comments/suggestions regarding the hackathon?	2.1 What did you learned from the hackathon one year after?	2.2 What has been the outcome of the hackathon on your work during this last year?	2.3 Did the hackathon helped you to organize similar events in your institution?	2.4 What kind of events did the hackathon helped you to organize during this last year?
A lot! We have been working together on-line or through social media for quite sometime to prepare for the hackathon, but the actual encounter and the real sessions where different teams get to collaborate and brain-storm have made the outcomes really amazing. I belong to the content team of precision medicine, therefore most -but not all- my participation was from a biologist/physician point of view. We learned what diseases that have the most detrimental impact in Africa, and hence they should be targeted the most in our search. Also, we learned about different resources that are publicly available and that are reputable enough for us to consider in linking them to our portal. We also learned how to perform manual curation for the data published in the literature. We agreed about simple definitions as race and ethnicity. We took important decisions as to use the ICD system for disease classification, and to include data describing Africans living outside the Continent and those with African ancestries in the database of the portal. We also realized certain gaps in the literature and decided to further study certain lagging areas; for instance the scarcity of data describing North Africa and the ambiguity in defining this part of Africa in the literature.	Collaboration, Enthusiasm, Friendly-atmosphere	Content team	African precision medicine portal	Socially: The preparation from our colleagues in Tunisia, and the outstanding hospitality. Scientifically: the brain-storming nature of session and the effective decision making process.	That we had to leave!	Virtual Hackathon would be great during this period of lockdown.	That continuous work is rewarding: we are almost ready to launch the precision medicine portal. We got some of the work published, and we are still working on the remaining. Therefore, I would say that perseverance and hard working (team working) is among the main things emphasized on, and maintained after the hackathon.	Coauthoring reviews and articles, participating in ebook writing, preparing for grant proposal in collaboration with scientists from different countries of the Continent.	No	I participate as a TA in the IBT 2020
I have learned team work, accountability and being proactive.<br>On the other hand I have learned about API, requirements to build a database and logical thinking	Highly organized, informative, productive.	Content team	African precision medicine portal	In general, I like the organization specially the feedback we provide by the end of each day and the follow up from central node.	The technical team faced some problems regarding the programming language to be used and this wasted few days of the hackathon	Increase the number of members of the technical team compared to the content team.	Commitment, because the hackathon was was just the begining.	I have been introduced to new databases, practiced data curation, design portals, I have learned how to critically evaluate my work.	Yes	H3ABioNet webinars
As this was my first hackathon, being a newcomer to such community, I never felt overwhelmed when it came to the project tasks, this is because of the camaraderie of team and how we solved problems together. Listening and be open to everyone's ideas is really an important outcome from hackathon. You may encounter blips at any points during the hackathon but it makes for a good memory particularly when you remember how the issues were overcome. It was a hackathon and training for me at the same time as I ended up increasing my experiment in developing database schema.	Experiences, New Knowledge, Widen Your Scope, Discipline But Fun , Achieving goals at a short time and Great Memory.	Content team	African microbiome portal	The friendly atmosphere	Can't think of any , was happy during this event particularly!	Providing steady Internet connection though I don't know how this can be tackled but this guarantee smooth running of most of the tasks and hence affect the final outcome.	My morale towards twisted issues has increased and become better iteratitacof myself, it also expanded my scope and drives me to accomplish things beyond my expectations.	Database schema design and critical evaluation of metadata filtering and curation paramerers needed to characterise African population	No	It helped me a lot in expanding my skills- coordination wise particularly during the last IBT and INBT (2019) courses!
Working Collaboratively	Fun, Interaction and Exploration	Technical team	Both portals	Interaction, people's different opinion on same topic and counter arguments. I also learnt, How leader make people work in coordination.	Too much sweets.	Basic pre-preparation on the topic. We spent almost 3 days discussing, which framework to choose.	What we achieve in hackathon in a day, takes a month afterward.	Exposure new web technologies, avoid reinventing wheel.	No	We didn't organize any hackathon.
Development skills	Great, Global, Amazing	Technical team	Both portals	The groups	There were only three competent developers in Python - Anmol, Alfred and Ziyaad. The rest were competent in other programming languages which did not help with the overall development deadlines and schedule.	Do proper due diligence on getting developers on board. Make sure they can code in the languages agreed upon. H3ABioNet is a Python development house.	I stopped working on it after a few months as I have not found the time to carry on with it.	If the current developers needed help I would assist them. Advised them on using PostgSQL and not NEO4J. Current developers are Anmol, Alfred and Michael Turkson.	No	Nothing
Some bibliography considérons microbiote	Team work	Content team	African microbiome portal	Team work	Duration	Nothing	Nothing	Abstract for events, drafting publications	No	No

1. 1 What did you learned during the hackathon held on April 2019 in Tunis?	1.2 Describe the hackathon in few words (3 max)	1.3 Which team you belonged to?	1.4 In which portal developmen t have you been involved?	1.5 What did you like the MOST about this hackathon?	1.6 What did you like the LEAST about this hackathon?	1.7 Any other comments/suggestion s regarding the hackathon?	2.1 What did you learned from the hackathon one year after?	2.2 What has been the outcome of the hackathon on your work during this last year?	2.3 Did the hackathon helped you to organize similar events in your institution?	2.4 What kind of events did the hackathon helped you to organize during this last year?
I learned a lot about team organisation, project design and how to divide up tasks for effective collaboration. I learned about issues like misclassification of North Africa in databases - which has implications for African researchers. I learned how to integrate different suggestions in order to synthesise a robust addition into what is desired. I learned concepts about database building and gained understanding into the types and methods used in such.	Communication is everything	Both teams	African precision medicine portal	The diversity of team-members and their expertise. We had members who individually are skilled, but worked well as a team to build a good project. I also liked that the members formed friendships that made for better collaboration after the hackathon. I enjoyed the setting and organisation of the hackathon which I feel helped us to achieve most of our objectives.	We started the hackathon on pre existing issues. It began with trying to use the "Catalog" code base, for which the original developer was not present, and we did not have anyone with the skill expertise to solve the pre-existing issues with that code. We also began with a change of concept - create API based databases which would access others in real time. We did not have this technical expertise present at the hackathon. We should have rather spent 2 days on designing the project in regard to strengths and weaknesses of our team members, and then moving to meet goals which are achievable in the hackathon time frame.	I think we were delayed most by the concept and goal of what we wanted out from the hackathon. We went into the hackathon after weeks of meetings and planning, but then changed our mind at the start of the hackathon. At this point, what we would need is a very open discussion about what we have the skill to achieve in the next few days. At the end of the hackathon though, we did have a good discussion about what we did manage to achieve, and how to go forward with progress.	I learned about the importance of being open and honest with your team about your skill, time constraints and what you are able to achieve. I also learned about the importance of concept design with critical feedback at the early stage. If we had begun with saying - we dont have the skill/time/resources to build an API based database, we may have reached our current trajectory sooner. It could be added that the current stage of the portal is not final. API based applications can still be added to the portal for later versions. I also learned about how important it is to share my own weaknesses and to report when you don't succeed, so that team members can help to get work back on track.	Learned a lot about database design and implementation, was able to learn more about the data and what limitations it had, had to reorganise the project back to a static based database in order to proceed - but the knowledge from the hackathon sped this up.	No	Good project design meetings for new researchers.
working as a team, sharing ideas and approaches	Interesting, very helpful, intensive work	Content team	African microbiome portal	being with all the members of the group in one place which was very helpful for improving our work on the portal	the planning of the hackathon was not clear before the event and we were discussing more the development before doing the conceptual model of the database	to have more hackathons	ideas about the importance of the portals in research	help me to contribute better in the microbiome portal work package	No	none
Working with an africain team	Team work	Technical team	African microbiome portal	The team	We dont have take time to finalize the conception before starting the developpement and during the selection stage, some programming language have been proposed such as php, html... to use them during the hackathon but during the event the programming language were changed twice and some of technical members did not have enough experience to work with these ones.	Nominate an IT project manager to manage the technical team.	Give us idea how to develop portal.	Starting to learn new progrmming language that used during the hackaton.	No	None
Working in a teamwork, following the SDLC and new programming skills.	Friendly teamwork, a great work environment and achievable	Technical team	Both portals	Managing and assigning the roles	The days are not enough	Nothing till now, we are doing a progress	Following the plan.	I learned how to work with alot of memebers in a teamwork, managing the roles and monitoring the progress.	No	Nothing till now
During the hackathon held on April 2019 in Tunis I learned programing using Python , Django , Java API and NOSQL Database.	Helpful, interesting and well organised	Technical team	African microbiome portal	Sharing of information between team members, team work	Not noticed.	It gets organized every year	Practice more the technologies learned during the hackathon.	It's benefit because I use what i have learned in my work ,<br>I made some relationships who help and guide me to solve some problems in my work.	Yes	The hackaton helped me to organise some training events to share my acquired knowledge with students .
